# Expression of cornulin in tongue squamous cell carcinoma

**DOI:** 10.3332/ecancer.2021.1197

**Published:** 2021-03-04

**Authors:** Saira Saleem, Iffat Aleem, Aribah Atiq, Sahrish Tariq, Amna Babar, Muhammad Abu Bakar, Madiha Syed, Maheen Maruf, Mohammad Tariq Mahmood, Muhammad Zeshan, Muhammad Tahseen, Raza Hussain, Asif Loya, Chris Sutton

**Affiliations:** 1 Basic Sciences Research, Shaukat Khanum Memorial Cancer Hospital and Research Centre, 7-A Block R-3, Johar Town, Lahore, 54000, Pakistan; 2 Department of Pathology, Shaukat Khanum Memorial Cancer Hospital and Research Centre, 7-A Block R-3, Johar Town, Lahore, 54000, Pakistan; 3 Cancer Registry and Clinical Data Management, Shaukat Khanum Memorial Cancer Hospital and Research Centre, 7-A Block R-3, Johar Town, Lahore, 54000, Pakistan; 4 Department of Surgical Oncology, Shaukat Khanum Memorial Cancer Hospital and Research Centre, 7-A Block R-3, Johar Town, Lahore, 54000, Pakistan; 5 Institute of Cancer Therapeutics, University of Bradford, Tumbling Hill Street Bradford, BD7 1BD, United Kingdom

**Keywords:** tongue squamous cell carcinoma, biomarker, manual tissue microarray, cornulin, decorin, collagen 1 alpha 2

## Abstract

The aim of the study is to identify cornulin (CRNN) protein expression associated with advancement of tongue squamous cell carcinoma (TSCC). A comparison of addictive (containing potential carcinogens) versus non-addiction causative agents was expected to allow detection of differences in CRNN expression associated with TSCC. Bespoke tissue microarrays (TMAs) were prepared and immunohistochemistry (IHC) performed to determine the changes in CRNN expression in epithelial cells of node-negative (pN-), node-positive (pN+) TSCC and non-cancer patients’ oral tissues. TMAs were validated by performing IHC on whole diagnostic tissues. Chi-square test or Fisher’s-exact tests were used to establish significant expression differences. Analogous analyses were performed for biomarkers previously associated with TSCC, namely collagen I alpha 2 (COL1A2) and decorin (DCN) to compare the significance of CRNN. Keratinisation and its level (low, extensive) were studied in relation to CRNN so that the extent of squamous differentiation could better be assessed.

IHC immunoreactive score (IRS) clustered the patients based on weak/moderate (Low (IRS ≤ +3)) or strong (High (IRS ≥ +4)) expression groups. A low expression was observed in a larger number of patients in control proteins COL1A2 (77.3%), DCN (87.5%) and target protein CRNN (52.3%), respectively. Low CRNN expression was observed in TSCC where nodes were involved (pN+: mean 1.4 ± 2.1) (*p* = 0.248). Keratinisation (%) was low (0% ≤ 50%) in 42.2% and extensive (1% ≥ 50.0%) in 57.8% patients. In conclusion, our study suggested that Low CRNN expression was associated with grade and lymph node metastasis in TSCC. CRNN expression is independent of addiction, however potentially carcinogenic addictive substances might be aiding in the disease progression.

## Introduction

According to GLOBOCAN, 354,864 (2.0% of all cancer sites) new cases and 177,384 (1.9% of all cancer sites) deaths/year were estimated worldwide, with South-Asia as a high-risk region, for lip and oral cavity cancers [1]. In the USA, 17,660 tongue cancer cases (males: 12,960; females: 4,700) and 2,830 deaths/year (males: 1,980; females: 850) were estimated [2]. In a study, the most common anatomical site (*n* = 44,072; 41%) was the tongue [3] while another group reported it to be third most affected site with buccal mucosa being first and mandibular alveolus as second [4]. Histological assessment of biopsy is a gold-standard of diagnosis of a malignancy. There is no biomarker for the early detection of any head and neck cancers, including tongue squamous cell carcinoma (TSCC) that could aid histological diagnosis of the disease. It is, therefore, imperative to improve knowledge on the proteins expressed in the tissue for earlier cancer detection and clinical management. This could be achieved by screening pre-cancerous and cancerous lesions of the mouth to recognise accurate signature protein biomarker(s). There have been a number of studies of TSCC, exploring single/panels of biomarkers of clinical relevance, though none were specific to squamous cells [5].

It is important to study specific protein expression in epithelial cells as SCC is mostly epithelial in origin and epithelial-to-mesenchymal transition is well understood. Cornulin (*CRNN*) gene discovered in human plays a role in epidermal differentiation [6] and its expression is believed to be specific to squamous cells [7]. Oral epithelium is prone to DNA damage which is further induced by habitual risk factors. CRNN functions as a stress-responsive factor in the buccal mucosa of tobacco-smokers [8]. Collagen I alpha 2 (COL1A2) is involved in carcinogenesis and modified expression of COL1A2 has been reported in association with several cancers [9–12]. Aberrant expression of decorin (DCN) in dysplastic and squamous carcinoma oral epithelial cells, but absent in normal mucosa tissue, has been reported suggesting its role as a possible biological marker of imminent progression [13].

In the present study, most of the tissues are from non-keratinising SCC, so CRNN was selected for validation particularly in epithelial cells as a marker of SCC differentiation. COL1A2 and DCN have been extensively studied and associated with TSCC, so their expression was considered as suitable references to assign significance to the expression of CRNN in the same sample set. In this study, we explore the expression of CRNN, COL1A2 and DCN in a well-characterised bank of non-cancer disease and pN^−^ and pN^+^ TSCC tissues with extensive records on use of addictive agents.

## Materials and methods

### Ethical statement

The Ethical approval for the study was obtained from the Institutional Review Board (IRB, registered with Office for Human Research Protections, USA; IORG0004939) at Shaukat Khanum Memorial Cancer Hospital and Research Centre (SKMCH&RC), Pakistan. All methods were carried out in accordance with the relevant guidelines [14]. A waiver from written consent was granted by the IRB at SKMCH&RC on grounds that the existing formalin-fixed paraffin-embedded (FFPE) tissues were used in the study and the anonymised results are reported ensuring patient confidentiality. The research involved no intervention/interaction with the participants with minimal risk and involved no procedures for which written consent is normally required.

### Patient selection

A retrospective study for tumour marker was planned [15]. A cohort of 176 patients (age range 15–80 years) was enrolled for biopsy (local/wide/wedge) of oral cavity and glossectomy with neck dissection at SKMCH&RC, Pakistan between January 2000 and August 2019. Of these patients, *n* = 128 were diagnosed with cancer and *n* = 48 were cancer-free. Of the cancer patients, some cancer TSCC (*n* = 43) could not be TNM staged because they underwent biopsy/glossectomy only without neck dissection and only grade (G1/G2/G3/G4) was reported except for two patients for which grade was not mentioned. The glossectomy with neck dissection cancer patients was TNM staged as well as graded. All analyses were made on tongue/oral cavity tissues. The surgeons assess to resect the nodes as required for the treatment of the patient based on the spread of the disease. Later on, all the resected nodes by the surgeon are examined for the presence of malignancy or reactive status. The nodes were examined for the presence of malignancy or reactive status by H&E stained tissues on slides. The nodes staging could only be provided for those patients who undergo neck dissection which is not the case for biopsy-only patients. The idea was to see the expression of biomarkers on tongue tissues so all archived tissues either removed via biopsy or glossectomy with neck dissection were included in the study because the aggressivity of the cancer is determined by its grade which is reported for all even if the neck nodes are not resected.

ll the patients were selected as per the study inclusion criteria (existing FFPE tissue blocks for patients with diagnosed TSCC or non-cancer oral cavity lesions) and study variables – addiction status (betel leaf/naswar/smoker addicts), cancer stage (I/II/III/IV), without previous anti-cancer radiation and/or chemotherapy. None of the patients had other malignancies in the past so never had chemo and radiation therapy for other malignancies. Only three TSCC had a previous history of SCC.

The patients having known history of HIV, HBV and HCV were excluded. A random non-cancer (biopsy-only) population representing group of non-cancer patients (*n* = 48) diagnosed with non-malignant mucosal diseases were also included as per study inclusion criteria in the study as a reference control. For this group, tissues were taken from the oral cavity sites (buccal mucosa, mouth floor, gum, lip, palate (*n* = 33) and tongue (*n* = 15)) with a reported histopathology (acute/chronic inflammation, cavernous haemangioma, granulation of squamous epithelium, hyperkeratosis, hyperplasia, parakeratosis or ulceration).

### Histopathology

The presence/absence of cancer cells in the acquired specimen was confirmed independently by two pathologists. The histopathological diagnoses and pathology (pTNM) staging of all cases of non-cancer and TSCC were confirmed following American Joint Committee on Cancer (AJCC) guidelines by the pathologists.

### Evaluation of keratinisation

The assessment of keratinisation was based on the H&E stained slides. The proportion of keratinisation in the entire diagnostic section was quantified as follows: Low 0% ≤ 50% and Extensive 1% ≥ 50%. Keratin pearls or keratin in individual cells were studied in the whole slide in case of squamous cell carcinoma.

### Construction of tissue microarray (TMA)

Donor block: H&E stained diagnostic sections from which patient’s histopathologic diagnosis was made, were used as a guide. A morphologically representative area of interest from each patient’s H&E stained archived slide was identified under the microscope and marked by the pathologists (AA, AB) by circling with permanent fine-point marker on the glass slide. Once the slides were reviewed and marked, the in-house ID Hospital information system number (HIS#) written on the glass slide was matched to HIS# written on the archived FFPE tissue block. The marked morphologically representative area of interest from each patient’s FFPE block (donor block) was identified. For the validation of TMAs, consecutive 3–4 µm sections from the donor blocks were cut for 80.0% of the patients and used for IHC staining for whole diagnostic tissue section slides.

Recipient array block: The TMA FFPE blocks (recipient) were constructed manually by relocating small cylindrical tissue cores (diameter: 2.0 mm diameter; length: 4 mm) [16] from patient’s FFPE donor block’s identified area and placing them in a TMA FFPE recipient block with defined array coordinates in a grid-like fashion with precise spacing (1 mm apart). Some other studies have reported TMA results on smaller tissue cores of <1 mm diameter [17, 18]. Of the 176 patient tissues, 23–26 tissues/recipient TMA block were used for the construction of TMA. A glass slide was moved in a circular motion on top of the TMA FFPE block and warmed at 35°–37°. The smooth TMA FFPE blocks were stored at −20°.

### Microtomy

Facing the array block on a microtome, 3–4 µm sections were cut and placed on positively-charged slides with careful orientation. TMA slides were prepared and used for H&E staining, COL1A2 IHC, CRNN IHC and DCN IHC.

### Immunohistochemistry (IHC)

IHC staining was conducted on the whole diagnostic sections (for validation) and TMA sections for the detection of expression of COL1A2, CRNN and DCN. After deparaffinisation in xylene and rehydration, for 10 minutes to retrieve antigenicity, slides were immersed in 3% hydrogen peroxide for 10 minutes at room temperature (RT) to block endogenous peroxidase activity, washed in distilled water and treated with microwave heating for 20 minutes in a Tris-EDTA buffer (pH 9.0), washed in distilled water and treated with phosphate-buffered saline (1×PBS) twice for 5 minutes. Incubation was carried out for 40 minutes at 37° with primary antibody (anti-COL1A2, rabbit polyclonal antibody, 1:200, N-terminal Keyhole Limpet Haemocyanin, Abcam, China, anti-CRNN rabbit polyclonal antibody, 1:200, 11799-1-AP, Proteintech, Chicago, USA and anti-DCN mouse monoclonal antibody, 1:200, 1–360 Amino Acid, Abcam, China). Visualisation was achieved by incubation with horseradish peroxidase-labelled secondary antibody (goat anti-rabbit or anti-mouse immunoglobulins) for 1 hour at RT, followed by the addition of 3, 30-diaminobenzidine as a chromogen to detect antigen–antibody complexes (EnVision, Dako, Carpinteria, USA). The tissues were counterstained with haematoxylin and dehydrated, cleared in xylene and mounted with Eukitt. Invasive ductal carcinoma of breast (lumpectomy), right buccal mucosa of inner cheek (excisional biopsy) and adrenocortical adenoma (adrenalectomy) served as a positive control for COL1A2, CRNN and DCN expression, respectively, and the primary antibody was replaced with 1×PBS for the negative control. The IHC evaluation was performed independently by pathologists (AA AB, MS and MM under the supervision of AL and MTM) in a blind study. All experiments were performed in duplicate to confirm scientific reproducibility. Olympus Deca head Microscope, model number BX53 with a mounted Olympus DP27 camera using Olympus cellSens Entry (1.18 build, 16686) imaging software was used to take images of the tissues.

#### Scoring of immunohistochemical staining

Immunopositive staining of COL1A2, CRNN and DCN was evaluated in the entire areas of the tissue sections independently by two pathologists. Sections were scored as positive if epithelial cells showed immunopositivity in the cytoplasm and/or nucleus when observed by the evaluators who were blinded to the clinical outcome. The scoring by two observers was discrepant in few cases and a consensus on the final result was reached by re-evaluation of these slides and discussion. An inter-rater reliability analysis using the Kappa-statistic was performed to determine consistency between evaluators.

#### Evaluation of IHC staining

**Supplementary Figure S1.**
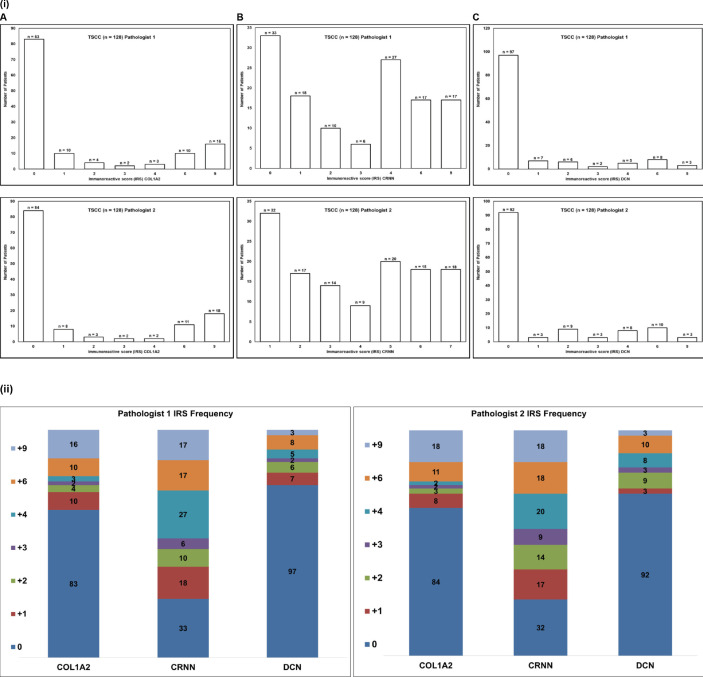
(i) Frequency histogram for the (A): COL1A2, (B): CRNN and (C): DCN. Immunoscore (IRS) cut-off value (+3). TSCC: Tongue squamous cell carcinoma, *n*: Number of patients. (ii) Stacked graph shows the number of patients showing IRS scores 0 to +9

The scoring assessment was based on the semi-quantitative immunoreactive score (IRS) by multiplying the positive intensity and the immunopositive cell percentage to obtain an IRS ranging from 0 to +9 [19, 20]. The positive intensity scores were quantified using the following scores: negative = 0, weak = 1, moderate = 2 and strong = 3. The proportion/percentage of immunopositive cells was quantified as follows: 0 = no staining, 1 ≤ 30%, 2 = 30%–60% and 3 ≥ 60% of positive epithelial cells. The combined IRS for each patient was recorded. Protein expression of COL1A2, CRNN and DCN was classified into two groups; Low (IRS ≤ +3) and High (IRS ≥ +4), respectively. There is no pre-established cut-off value for COL1A2, CRNN and DCN so the frequency histograms for IRS were evaluated based on earlier studies [19] (Supplementary Figure S1A–C, respectively).

### Statistical analysis

Low (IRS ≤ +3) versus High (IRS ≥ +4) COL1A2, CRNN and DCN expression of oral tissues/tumours was considered to be one of the microscopic outcomes of the patients. The clinical outcome was the difference in COL1A2, CRNN and DCN expression based on addiction (betel leaf/naswar/tobacco smoke) among the cancer group. A cut-off value (+3) of candidate protein biomarker CRNN and reference protein biomarkers COL1A2 and DCN was chosen for defining positivity (Low/High score). Statistical analysis was carried out using the SPSS software (version 20.0; SPSS, Chicago, IL, USA). Continuous variables were stated as mean ± standard deviation (SD) and categorical variables were computed as frequencies and percentages. The continuous variables were compared using the independent *t*-test. Categorical variables were compared using the Chi-square test or Fisher’s exact test (when necessary) to assess the associations among categorical variables (COL1A2, CRNN and DCN protein expression and the clinico-pathological parameters). Statistical significance was defined as a two-tailed *p*-value < 0.05.

## Results

### Clinical association of COL1A2, CRNN and DCN in TSCC and non-cancer patients

The socio-demographical and baseline clinico-pathological characteristics of TSCC patients (*n* = 128), recruited for IHC study, including age, site of lesion, histopathological grade are presented in Table 1. Based on gender, 61.9% (*n* = 109) of the patients were male constituting larger proportion of the study cohort, while 38.1% (*n* = 67) were females. The mean age at diagnosis and SD for both male and female patients (*n* = 176) was 47.5 ± 14.4 years. The inter-rater reliability for the evaluators was found to be K = 0.86 (*p* < 0.001, 95% CI = 0.77–0.95) for all the cases *n* = 176 (cancer and non-cancer).

**Supplementary Figure S2.**
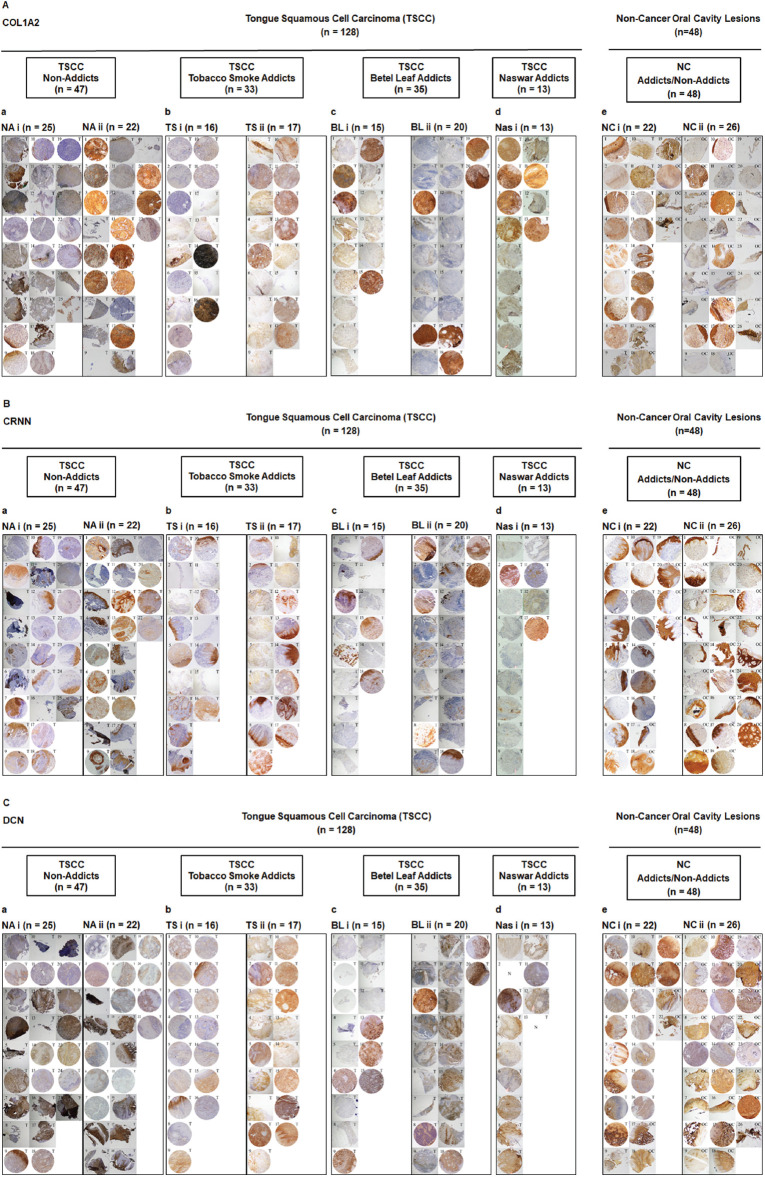
Representative images showing TMA for (A): COL1A2, (B): CRNN and (C): DCN IHC staining. a: Group of TSCC patients with no addiction (TSCC, NA). b: Group of TSCC patients who are tobacco smoke addicts (TSCC, TS). c: Group of TSCC patients who are betel leaf addicts (TSCC, BL). d: Group of TSCC patients who are naswar addicts (TSCC, Nas). e: Non-cancer control oral cavity tissues/lesions. (i and ii) Two TMA blocks per group. One image represents one patient. Image annotation: (N) Not available, (T) Tongue tissue (magnification, ×40).

**Figure 1.**
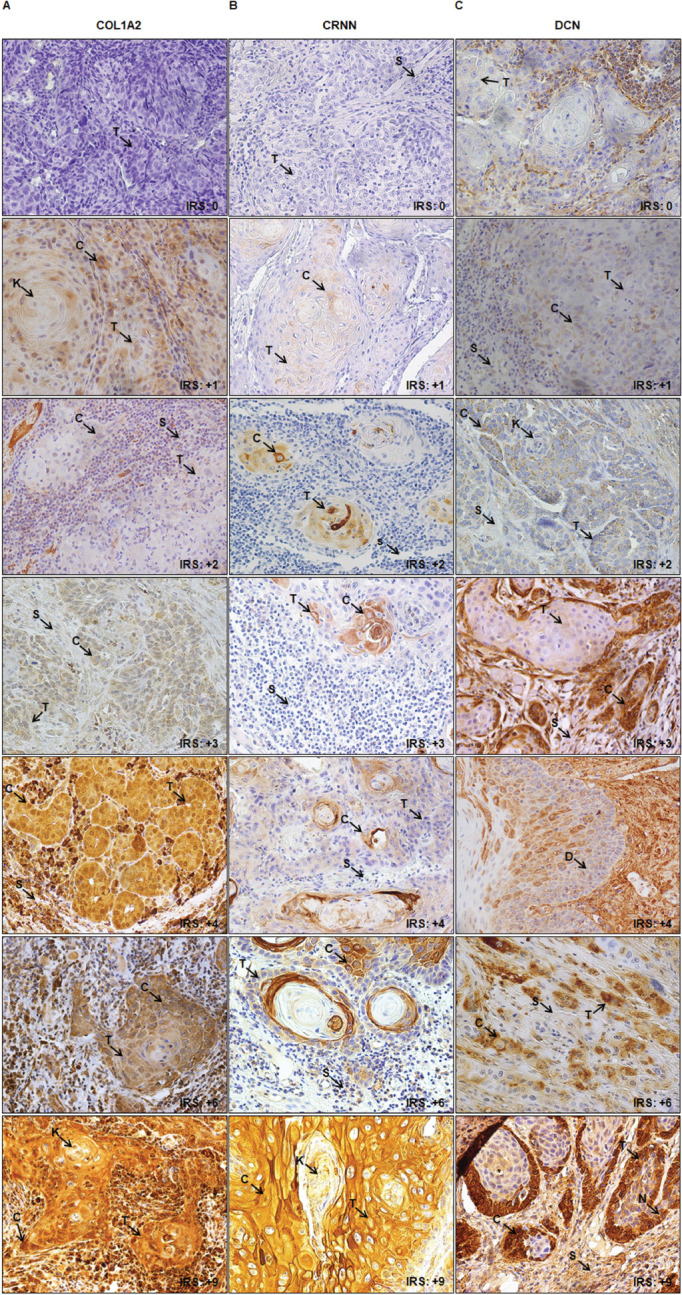
Representative images of IHC IRS score (0 to +9) in epithelial cells of TSCC. (a): COL1A2. (b): CRNN. (c): DCN. Image annotations: (C) Cytoplasmic staining, (K) Keratin crystal, (S) Stroma, (T) Tumour (magnification, ×40).

Representative images for the IHC IRS score 0 to +9 for COL1A2, CRNN and DCN are shown in Figure 1a–c, respectively. Representative observations of the IHC stains for COL1A2, CRNN and DCN for all patients’ TMA expression are shown in Supplementary Figure S2A–C respectively. Same IRS scores for the expression of COL1A2, CRNN and DCN were observed by whole diagnostic tissue section and 2.0 mm core TMA (representative of the whole section), validating the TMA method. Cytoplasmic COL1A2 was observed in all COL1A2 positive (Low or High). Based on the immunohistochemical score (cut-off at +3), there was a distribution in COL1A2 expression in all cases (*n* = 176) which separated patients into 77.8% with Low COL1A2 expression and 22.2% with High COL1A2 expression (Supplementary Table S1A). Cytoplasmic CRNN was observed in all positive cases (Low or High), but no detectable expression was observed in nucleus except two patients (due to intra-observer variation between the two pathologists). There was a clear binary distribution in CRNN expression in all cases (*n* = 176) which separated patients into 39.8% (*n* = 70) with Low CRNN expression and 60.2% (*n* = 106) with High CRNN expression (Supplementary Table S1B). Cytoplasmic DCN was observed in all DCN positive (Low or High), but expression was observed in nucleus in six patients. All 176 patient tissues were divided into Low DCN expression 156 (88.6%) and High DCN expression 20 (11.4%) (Supplementary Table S1C).

**Supplementary Figure S3.**
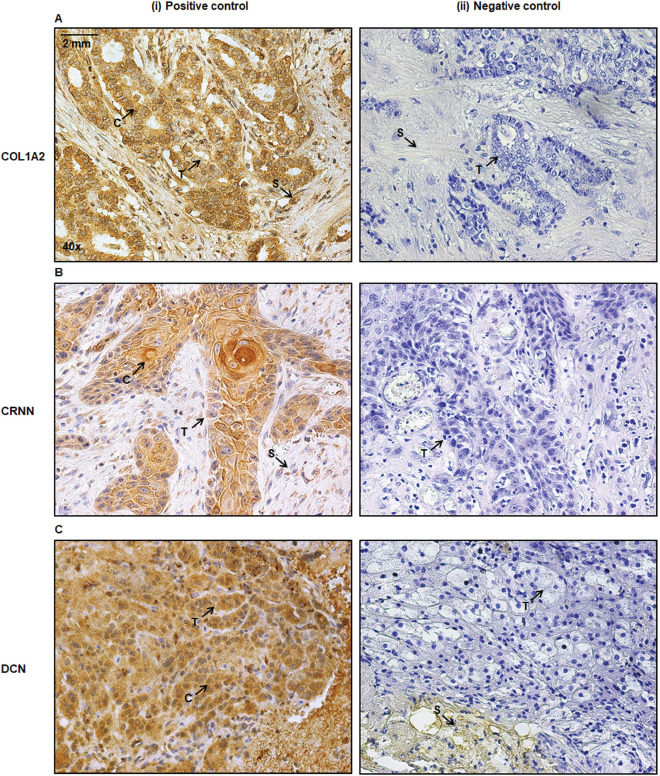
(A): Breast tumour cells are positive control (i) for anti-COL1A2 antibody. (B): Buccal mucosa of inner cheek as a positive control (i) for anti-CRNN antibody. (C): Adrenocortical adenoma cells are positive control (i) for anti-DCN. The primary antibody was replaced with 1×PBS for the negative control (ii). Image annotations: (C) Cytoplasm, (S) Stroma, (T) Tumour (magnification, ×40).

**Figure 2.**
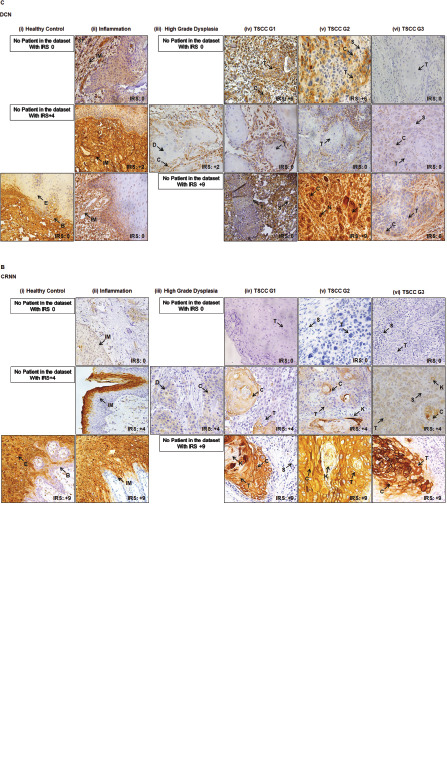
Representative images showing healthy and TSCC tissues. (a): COL1A2. (b): CRNN. (c): DCN IHC cytoplasmic and nuclear staining in epithelial cells of tongue. (i) Healthy tongue tissue control. (ii) Inflammation of tongue. (iii) High-grade dysplasia of tongue. (iv) Well differentiated (TSCC G1). (v) Moderately differentiated (TSCC G2). (vi) Poorly differentiated (TSCC G3). One image represents one patient. Image annotations: (B) Basal layer, (C) Cytoplasmic staining, (D) Dysplasia, (E) Epithelium, (IM) Inflammatory mucosa, (K) Keratin crystal, (N) Nuclear staining, (S) Stroma, (T) Tumour (magnification, ×40).

COL1A2, CRNN and DCN showed a degree of variation in its expression in healthy mucosa, inflammation, high grade dysplasia and well-poorly differentiated TSCC (Figure 2a–c). Representative images of negative and positive controls for anti-COL1A2, anti-CRNN and anti-DCN antibodies are shown in Supplementary Figure S3A–C. In all non-cancer oral mucosal tissues, epithelial cells showed strong (IRS = +9) cytoplasmic staining with anti-CRNN antibody in the epithelium (Supplementary Figure S3B).

### Clinical association of COL1A2, CRNN and DCN in TSCC

All the statistical analyses described here were calculated on the 128 TSCC patients (Table 1). COL1A2 expression did not correlate significantly with the tumour stage, lymph node, unknown metastasis and tumour grade (Table 1A). A greater proportion of TSCC patients with positive CRNN expression had stage T1–T2 tumours (60%). On the basis of histopathological characteristics, these TSCC were sub-classified as G1/well differentiated (46 patients), G2/moderately differentiated (69 patients) and G3/poorly differentiated (9 patients) carcinoma and showed Low CRNN expression (*p* = 0.02) (Table 1B). Patients (52.3%) with Low CRNN expression had largest tumour greatest dimension (cm) (mean 2.4 ± 1.1 cm) than those with High CRNN expression (47.7%) (mean 1.8 ± 1.2 cm; *p* = 0.027) (Table 1B). Low and High DCN expression was noted; 87.5% and 12.5%, respectively (Table 1C). DCN expression did not correlate significantly with any of the clinical parameters (Table 1C).

### Effect of addictions

**Figure 3.**
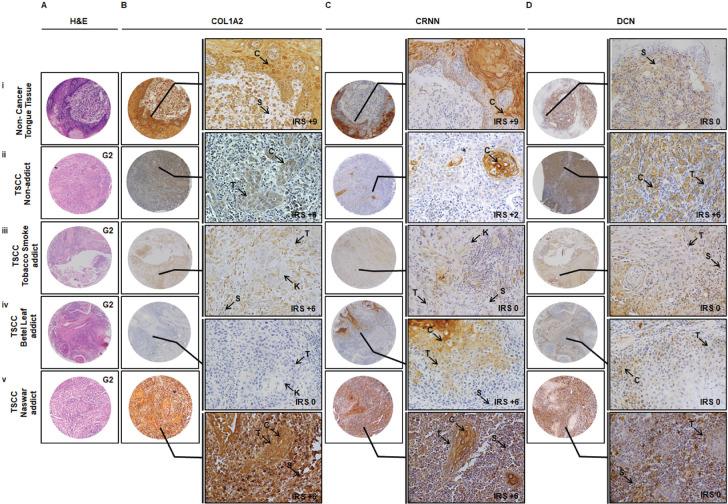
Representative images showing TMA Immunohistochemical association of COL1A2, CRNN and DCN in non-cancer oral lesions and TSCC in terms of addictive agents. IHC was carried out in tissue sections using specific antibody for COL1A2, CRNN and DCN as described in Material and Methods section. (a): H&E staining. (b): Immunostaining of CRNN in (i) non-cancer tongue tissue, (ii) TSCC, non-addict, (iii) TSCC, tobacco smoke addict, (iv) TSCC, betel leaf addict and (v) TSCC, naswar addict. Image annotations: , (C) Cytoplasmic staining, (IM) Inflammatory mucosa, (K) Keratin crystal, (S) Stroma, (T) Tumour, (IRS) IRS, (0 to +9) score to show gradient expression (TMA and inset: magnification, ×40).

Representative observations of the H&E staining, immunohistochemical stains for COL1A2, CRNN and DCN in the same patient each are shown in Figure 3a–d, respectively. COL1A2 expression did not correlate significantly with the addictive agents betel leaf (*p* = 0.418) (Table 2A(i)) and naswar (*p* = 0.522) (Table 2A(ii)) however, correlated significantly with addictive agent tobacco smoke in TSCC (*p* = 0.001) (Table 2A(iii)).

CRNN expression did not correlate significantly with addictive agent betel leaf (*p* = 0.091) (Table 2B(i)) and addictive agent tobacco smoke in TSCC (*p* = 0.150) (Table 2B(iii)). CRNN expression significantly correlated with addictive agent naswar in TSCC (*p* = 0.003) (Table 2B(ii)). DCN expression did not correlate significantly with any addictive agent betel leaf (*p* = 0.737) (Table 2C(i)), addictive agent naswar (*p* = 0.182) (Table 2C(ii)) and addictive agent tobacco smoke in TSCC (*p* = 0.511) (Table 2c(iii)). As for COL1A2 and CRNN, a similar pattern is evident in case of DCN that a higher number TSCC patients who use addictive agents betel leaf, naswar or tobacco smoke show Low DCN expression, respectively. Non-addicts also show the opposite pattern with Low IRS. Overall, 63% TSCC patients were addicts and 37% were non-addicts and correlated significantly with COL1A2 (*p* = 0.019) and CRNN (*p* = 0.015) (Supplementary Table S2).

### Clinical association of COL1A2, CRNN and DCN in non-cancer patients

Since this is a single-centre study, all the existing FFPE tissue blocks were used for the non-malignant group representing a random population without an oral malignancy. Patients who did not provide information on substance usage (i.e. stated ‘unknown’ in the questionnaire) were most likely not using any addictive agents. All the non-cancer patients were biopsy-only patients so nodes were not resected/studied. Cytoplasmic COL1A2, CRNN and DCN expression in epithelial cells was studied in 50.0% (*n* = 24) of the male patients, while 50.0% (*n* = 24) were females. A High CRNN cytoplasmic expression was observed in 45 of the 48 (93.8%) non-cancer patients (Table 3B). On the contrary, both reference marker proteins COL1A2 and DCN showed a Low cytoplasmic expression in 38 (79.2%) and 44 (91.7%) non-cancer patients (Table 3A and C). Data analysis (Tables 1–3) from Pathologist 2 is shown in Supplementary Table S3.

Cardinal percentages of immunopositive cells and keratinisation in TSCC and non-cancer without categorising data based on cutoff point is shown in Supplementary Table S4.

## Discussion

The manuscript describes research in normal and cancer biopsies of a specific sub-region, i.e. tongue, hence this removes a potential variation in the interpretation to enable a clearer understanding of CRNN expression. In the present study, we made TMAs from TSCC and non-cancer (healthy control patient with a lesion that was non-malignant and had no other remarkable study to distinguish) and effects on COL1A2, CRNN and DCN expression as a result of different addictive agents (naswar, betel leaf and tobacco smoking). The findings were further correlated with clinico-pathological variables. According to our findings, CRNN expression was high (93.8%, Table 3B) in the non-malignant oral tissues but was reduced or absent in the primary TSCC tissue specimens and varied based on addiction status but was reduced in cases where cause was not recorded or unknown. This is in agreement with the previous studies in which CRNN expression has been reported to differentiate between low-grade and high-grade oral epithelial dysplasia and could be represented as a potential biomarker for the assessment of progression of oral cancers [21]. Up-regulated CRNN levels prevent lesion formation and its tumour suppressive role has been reported [22]. *CRNN* was found significantly downregulated in TSCC in a genome-wide transcriptomic study on 53 primary tumour tissues [23]. An IRS score (0 to +9) to reflect CRNN IHC cytoplasmic expression in epithelial cells to use in addition to histologic analyses of TSCC. Keratinisation levels were studied in relation to CRNN. Cytokeratins (CKs) have been reported to be indicative of squamous cell differentiation [24]. Keratin 4 (CK4) is a marker of dysregulation of oral epithelial differentiation [25], CK10 expressed strongly in squamous cells [26], CK14 fell as the severity of the disease progressed from low- to high-grade dysplasia to SCC [27]. CK1/10 are markers for keratinised epithelium and CK4/13 can be used as markers of non-keratinised epithelium [28]. *CRNN* is believed to be a marker of keratinocyte proliferation [29]. CK19 expression has been reported in dysfunctional oral epithelial differentiation [30, 31] and a biomarker of highly invasive oral squamous cell carcinoma with metastatic potential [32].

Both COL1A2 and DCN have been reported to be expressed mainly in extracellular spaces extracellular matrix [33, 34], in extracellular membranes or as secreted proteins. We studied their expression in epithelial cells particularly and calculated an IRS to semi-quantify the expression levels of the COL1A2 and DCN protein in normal and tongue cancer cases for comparison with CRNN. Our findings of substantially decreased expression in epithelial cells and tumour associated connective tissues are in line with the animal model studies where genetic deletion of DCN facilitates intestinal tumour formation [35]. Research shows that three proteins, namely CCL13 (chemokine), DCN (an inhibitory protein) and Interleukin6 (cytokine) participate in more than 75% for the prediction of cell proliferation [36]. DCN is also expressed and secreted by fibroblasts in the stroma where it binds to collagen I stimulatory surfaces to antagonise tumour growth by overcoming TGF-β-mediated immunosuppression [37, 38], suggesting an important role in stroma-cancer cell communication [39, 40].

Cancer originating in epithelial layer invades sub-epithelial layer by degrading basement membrane (BM) leading to metastasis. Collagens are the structural components of BM maintaining its integrity and function. Loss of cell contour has been associated with cell transformation and metastasis [41]. The altered expression of COL1A2 in epithelial cells correlates with various structural changes in tongue during disease progression. The hyper-proliferative neoplastic cells may induce COL1A2 degradation to facilitate tumour invasion. As all three proteins (COL1A2, CRNN and DCN) are decreasing in epithelial cells, this means these have a positive correlation. Destruction of structural protein COL1A2, and loss of tumour related proteins CRNN and DCN support the speculation that all three are involved in a pathway for TSCC genesis. The findings summarised in this study indicate that TSCC development is an addiction independent event; however, this invites further investigations to find effects of chemical components of various addiction substances (betel leaf, naswar and tobacco smoke). In a meta-analysis of eight microarray studies, selected biomarkers were subdivided into +q6 and −q6 that correlated to two recognised high-risk HNSCC populations. The −q6 group (six genes including *CRNN*) were younger, female, betel quid-chewers [42]. For all the tested parameters, a similar response was observed between CRNN and DCN (Table 4).

CRNN can be supplementary to H&E histological assessment based on the degree of variation in its expression. The IRS can be different in TSCC patients with the same grade (G2) based on various addictions (Figure 3). Reduced expression in oral SCC associated with poor prognosis reflected that *CRNN* might not have the ability to respond to DNA damage induced by habitual risk-factors of smoking/alcohol/betel-quid [43]. Additional investigation of the underlying mechanism of CRNN downregulation is critical to defining how CRNN contributes to TSCC carcinogenesis and how its expression varies with various risk-factors such as betel leaf, naswar, tobacco and other addiction carcinogenic substances. The clinical implications of CRNN expression should be validated in a larger clinical cohort to establish its role as a prognostic and diagnostic marker for neck-node negative or neck-node positive TSCC.

We propose that scores, such as IRS, for biomarkers described here for epithelial cells should be calculated for major cell populations within a tissue, its extracellular matrix and spaces to calculate an aggregate index and use that for the diagnosis and prognosis of certain cancer. Non-malignant and malignant tissues were selected to observe subtle changes of a known biomarker, CRNN, using IHC at molecular level on anatomically-specific tongue tissue based on the addiction status. This is the first study to analyse the co-expression and positive correlation between structural protein COL1A2, tumour related protein CRNN and collagen binding protein DCN; however, further research is required to investigate these observations. Our findings served only to confirm seemingly contradictory evidence of CRNN increase and decrease in TSCC that has been reported previously. Our study was limited by the number of addiction-wise matched patients in non-cancer population, nevertheless, our results support the clinical usefulness of CRNN as a potential biomarker in clinical practice in future as an IHC tool to stratify malignancy.

## Conclusions

A loss/decrease or gain/increase in the expression of a certain marker could be attributed to the mere result of consumption of a certain chemical component from one of these addictions (betel nut/naswar/tobacco smoke) which might lead to incorrect diagnosis. We affirm the role of CRNN in development of TSCC; however, the experimental evidence to confirm the effects of causative agents on its expression remains to be determined.

## Conflicts of interest

The authors declare that they have no conflicts of interest.

## Authors’ contributions

SS: conception and design of the study, acquisition of data, analysis and interpretation of data, drafting the article, revising it critically for important intellectual content, final approval of the version to be submitted. IA: acquisition of data, analysis and interpretation of data, drafting the article, final approval of the version to be submitted. AA, ST, AB, MS, MM: acquisition of data, analysis and interpretation of data, final approval of the version to be submitted. MTM: acquisition of data, analysis and interpretation of data, revising the draft critically for important intellectual content, final approval of the version to be submitted. MZ: acquisition of data, analysis of data, drafting the article, final approval of the version to be submitted. MT: acquisition of data, analysis of data, final approval of the version to be submitted. MAB: statistical analysis. RH, AL: acquisition of data, analysis and interpretation of data, revising the draft critically for important intellectual content, final approval of the version to be submitted. CS: study design, interpretation of data, drafting article. All approved manuscript.

## Funding

This research did not receive any specific grant from funding agencies in the public, commercial or not-for-profit sectors. This work was supported by SKMCH&RC, Pakistan. The sponsor was not involved in the study design; in the analysis and interpretation of the data; in the writing of the report or in the decision to submit the paper for publication.

## Institutional ethics approval

The study approval was obtained from the IRB at SKMCH&RC, Lahore, Pakistan. The relevant guidelines (Declaration of Helsinki) and regulations were followed. This has been stated in the Materials and methods section.

## Patient consent for publication

The waiver of the consent of all participating subjects is obtained from the IRB at SKMCH&RC, Pakistan.

## Data availability statement

The raw data file for the TMA is supplied as Supplementary Material. The deidentified data set for this study is available upon request from SKMCH&RC.

## Consent for publication

The material submitted is new, original, in accordance with ICMJE and has not been submitted to another journal for concurrent consideration. All co-authors agree for publication upon acceptance and we agree on copyright ownership for the article as per Journal policy. We request for full waiver of article processing charges for publication as the research is from lower middle income country Pakistan and no external grant is secured.

## Figures and Tables

**Supplementary Table S1.** Association between clinico-pathological features of TSCC and non-cancer (A) COL1A2, (B) CRNN and (C) DCN immunohistochemical staining.

A			
Pathologist 1	COL1A2 Expression		*p*-value
	Low^a^	High^b^	
**TSCC and Non-Cancer Patients (*n* = 176)**	137 (77.8%)	39 (22.2%)	—
Age at Diagnosis (years)			
mean ± SD	47.7 ± 14.7	46.82 ± 14.3	0.733
**Gender**			
Male (*n* = 109)	91 (83.5%)	18 (16.5%)	*0.021
Female (*n* = 67)	46 (68.7%)	21 (31.3%)	

Pathologist 2	COL1A2 Expression		*p*-value
	Low^a^	High^b^	
**TSCC and Non-Cancer Patients (*n* = 176)**	132 (75.0%)	44 (25.0%)	—
**Age at Diagnosis (years)**			
mean ± SD	48.0 ± 14.4	46.1 ± 14.5	0.442
**Gender**			
Male (*n* = 109)	89 (81.7%)	20 (18.3%)	*0.009
Female (*n* = 67)	43 (64.2%)	24 (35.8%)	

B			
Pathologist 1	CRNN expression		*p*-value
	Low^a^	High^b^	
**TSCC and Non-Cancer Patients (*n* = 176)**	70 (39.8%)	106 (60.2%)	—
**Age at Diagnosis (years)**			
mean ± SD	48.1 ± 13.3	47.11 ± 15.1	0.648
**Gender**			
Male (*n* = 109)	52 (47.7%)	52 (52.3%)	*0.006
Female (*n* = 67)	18 (26.9%)	49 (73.1%)	

Pathologist 2	CRNN Expression		*p*-value
	Low^a^	High^b^	
**TSCC and Non-Cancer Patients (*n* = 176)**	75 (42.6%)	101 (57.4%)	—
**Age at Diagnosis (years)**			
mean ± SD	48.0 ± 13.5	47.1± 15.1	0.679
**Gender**			
Male (*n* = 109)	57 (52.3%)	52 (47.7%)	*0.001
Female (*n* = 67)	18 (26.9%)	49 (73.1%)	

C			
Pathologist 1	DCN Expression		*p*-value
	Low^a^	High^b^	
**TSCC and Non-Cancer Patients (*n* = 176)**	156 (88.6%)	20 (11.4%)	—
**Age at Diagnosis (years)**			
mean ± SD	47.1 ± 14.3	50.5 ± 14.9	0.326
**Gender**			
Male (n = 109)	93 (85.3%)	16 (14.7%)	0.077
Female (n = 67)	63 (94.0%)	4 (6.0%)	

Pathologist 2	DCN Expression		*p*-value
	Low^a^	High^b^	
**TSCC and Non-Cancer Patients (*n* = 176)**	155 (88.0%)	21 (12.0%)	—
**Age at Diagnosis (years)**			
mean ± SD	47.0 ± 14.3	52.3 ± 14.32	0.102
**Gender**			
Male (*n* = 109)	94 (86.2%)	15 (13.8%)	0.34
Female (*n* = 67)	61 (91.0%)	6 (9.0%)	

COL1A2, Collagen I alpha 2; CRNN, Cornulin; DCN, Decorin; TSCC, Tongue squamous cell carcinoma; SD, Standard deviation

*p*-value, Pearson’s chi-square test; pTNM and stage, AJCC guidelines

a IRS ≤ +3

b IRS ≥+ 4

* significant values (≤0.05)

**Supplementary Table S2.** TSCC addicts (betel leaf/naswar/tobacco smoke) versus TSCC non-addicts. (A): collagen I alpha 2 (COL1A2), (B): Cornulin (CRNN) and (C): Decorin (DCN).

A				
Pathologist 1		COL1A2 expression		*p*-value
		Low^a^	High^b^	
**TSCC**	Addicts (*n* = 81)	68 (84.0%)	13 (16.0%)	*0.019
	Non-Addicts (*n* = 47)	31 (66.0%)	16 (34.0%)	
**Total**	**128**	99 (77.3%)	29 (22.7%)	

Pathologist 2		COL1A2 expression		*p*-value
		Low^a^	High^b^	
**TSCC**	Addicts (*n* = 81)	66 (81.5%)	15 (18.5%)	*0.048
	Non-Addicts (*n* = 47)	31 (66.0%)	16 (34.0%)	
**Total**	**128**	97 (75.8%)	31 (24.2%)	

B				
Pathologist 1		CRNN expression		*p*-value
		Low^a^	High^b^	
**TSCC**	Addicts (*n* = 81)	49 (60.5%)	32 (39.5%)	*0.015
	Non-Addicts (*n* = 47)	18 (38.3%)	29 (61.7%)	
**Total**	**128**	67 (52.3%)	61 (47.7%)	

Pathologist 2		CRNN expression		*p*-value
		Low^a^	High^b^	
**TSCC**	Addicts (n = 81)	53 (65.4%)	28 (34.6%)	*0.006
	Non-Addicts (n = 47)	19 (40.0%)	28 (59.6%)	
**Total**	**128**	38 (63.3%)	22 (36.7%)	

C				
Pathologist 1		DCN expression		*p*-value
		Low^a^	High^b^	
**TSCC**	Addicts (*n* = 81)	73 (90.1%)	8 (9.9%)	0.239
	Non-Addicts (*n* = 47)	39 (83.0%)	8 (17.0%)	
**Total**	**128**	112 (87.5%)	16 (12.5%)	

Pathologist 2		DCN expression		*p*-value
		Low^a^	High^b^	
**TSCC**	Addicts (*n* = 81)	70 (86.4 %)	11 (13.6 %)	0.257
	Non-Addicts (*n* = 47)	37 (78.7%)	10 (21.3%)	
**Total**	**128**	107 (83.6%)	21 (16.4%)	

COL1A2, Collagen I alpha 2; CRNN, Cornulin; DCN, Decorin; TSCC, Tongue squamous cell carcinoma

*p*-value, Pearson’s chi-square test; ^1, 2 and 3^TSCC Nodes not resected, Node negative and Node positive patient numbers

a IRS ≤ +3

b IRS ≥ +4

* Significant values (≤ 0.05)

**Supplementary Table S3.** Data analysis (Table 1-3 in manuscript) from Pathologist 2.

A			
Pathologist 2	COL1A2 expression		*p*-value
	Low^a^	High^b^	
**TSCC Patients (*n*** = **128)**	97 (75.8%)	31 (24.2%)	—
**Age at Diagnosis (years)**			
mean ± SD	49.4 ± 13.1	49.3 ± 13.8	0.971
**Gender**			
Male (*n* = 85)	72 (84.7%)	13 (15.3%)	*0.001
Female (*n* = 43)	25 (58.1%)	18 (41.9%)	
**Pathological T Stage (pT)**			
pT1 (*n* = 47)	36 (76.6%)	11 (23.4%)	0.536
pT2 (*n* = 30)	25 (83.3%)	5 (16.7%)	
pT3 (*n* = 8)	5 (62.5%)	3 (37.5%)	
pT4 (*n* = 0)	0	0	
NA (*n* = 43)	31 (72.1%)	12 (27.9%)	
**Pathological N Stage (pN)**			
pNX (*n* = 11)	6 (54.4%)	5 (45.5%)	0.153
pN0 (*n* = 40)	34 (85.0%)	6 (15.0%)	
pN1 (*n* = 21)	14 (66.7%)	7 (33.3%)	
pN2b (*n* = 12)	11 (91.7%)	1 (8.3%)	
pN2c (*n* = 1)	1 (100%)	0	
NA (*n* = 43)	31 (72.1%)	12 (27.9%)	
**Pathological M Stage (pM)**			
pMX (*n* = 85)	66 (77.6%)	19 (22.4%)	0.488
NA (*n* = 43)	31 (72.1%)	12 (27.9%)	
**Stage (pTNM)**			
I (*n* = 30)	23 (76.7%)	7 (23.3%)	0.51
II (*n* = 17)	14 (82.4%)	3 (17.6%)	
III (*n* = 25)	17 (68.0%)	8 (32.0%)	
IV (*n* = 13)	12 (92.3%)	1 (7.7%)	
NA (*n* = 43)	31 (72.1%)	12 (27.9%)	
**Grade**			
G1 (*n* = 46)	37 (80.4%)	9 (19.6%)	0.428
G2 (*n* = 69)	52 (75.4%)	17 (24.6%)	
G3 (*n* = 9)	6 (66.7%)	3 (33.3%)	
G2-G3 (*n* = 2)	1 (50.0%)	1 (50.0%)	
NA (*n* = 2^¥^)	1 (50.0%)	1 (50.0%)	
**Tumor Site**			
Tongue (*n* = 128)	97 (75.8%)	31 (24.2%)	—
**Tumor Greatest Dimension (cm)**			
mean ± SD	2.2 ± 1.1	1.9 ± 1.3	0.326
**Nodes Involved**			
mean ± SD	1.2 ± 1.8	1.1 ± 2.2	0.937

B			
Pathologist 2	CRNN expression		*p*-value
	Low^a^	High^b^	
**TSCC Patients (*n*** = **128)**	72 (56.2%)	56 (43.8%)	—
**Age at Diagnosis (years)**			
mean ± SD	48.6 ± 12.8	50.4 ± 13.9	0.442
**Gender**			
Male (*n* = 85)	54 (63.5%)	31 (36.5%)	*0.02
Female (*n* = 43)	18 (41.9%)	25 (58.1%)	
**Pathological T Stage (pT)**			
pT1 (*n* = 47)	24 (51.1%)	23 (48.9%)	0.342
pT2 (*n* = 30)	21 (70.0%)	9 (30.0%)	
pT3 (*n* = 8)	5 (62.5%)	3 (37.5%)	
pT4 (*n* = 0)	0	0	
NA (*n* = 43)	22 (51.2%)	21 (48.8%)	
**Pathological N Stage (pN)**			
pNX (*n* = 11)	6 (54.5%)	5 (45.5%)	0.397
pN0 (*n* = 40)	21 (52.5%)	19 (47.5%)	
pN1 (*n* = 21)	12 (57.1%)	9 (42.9%)	
pN2b (*n* = 12)	10 (83.3%)	2 (16.7%)	
pN2c (*n* = 1)	1 (100%)	0	
NA (*n* = 43)	22 (51.2%)	21 (48.8%)	
**Pathological M Stage (pM)**			
pMX (*n* = 85)	50 (58.8%)	35 (41.2%)	0.409
NA (*n* = 43)	22 (51.2%)	21 (48.8%)	
**Stage (pTNM)**			
I (*n* = 30)	15 (50.0%)	15 (50.0%)	0.265
II (*n* = 17)	10 (58.8%)	7 (41.2%)	
III (*n* = 25)	14 (56.0%)	11 (44.0%)	
IV (*n* = 13)	11 (84.6%)	2 (15.4%)	
NA (*n* = 43)	22 (51.2%)	21 (48.8%)	
**Grade**			
G1 (n = 46)	31 (67.4%)	15 (32.6%)	0.348
G2 (n = 69)	34 (49.3%)	35 (50.7%)	
G3 (*n* = 9)	5 (55.6%)	4 (44.4%)	
G2-G3 (*n* = 2)	1 (50.0%)	1 (50.0%)	
NA (*n* = 2^¥^)	1 (50.0%)	1 (50.0%)	
**Tumor Site**			
Tongue (*n* = 128)	72 (56.2%)	56 (43.8%)	—
**Tumor Greatest Dimension (cm)**			
mean ± SD	2.4 ± 1.2	1.7 ± 1.1	*0.005
**Nodes Involved**			
mean ± SD	1.4 ± 2.1	0.8 ± 1.5	0.174

C			
Pathologist 2	DCN expression		*p*-value
	Low^a^	High^b^	
**TSCC Patients (*n* = 128)**	107 (83.6%)	21 (16.4%)	—
**Age at Diagnosis (years)**			
mean ± SD	48.8 ± 13.0	52.3 ± 14.2	0.263
**Gender**			
Male (*n* = 85)	70 (82.4%)	15 (17.6%)	0.594
Female (*n* = 43)	37 (86.0%)	6 (14.0%)	
**Pathological T Stage (pT)**			
pT1 (*n* = 47)	39 (83.0%)	8 (17.0%)	0.335
pT2 (*n* = 30)	25 (83.3%)	5 (16.7%)	
pT3 (*n* = 8)	5 (62.5%)	3 (37.5%)	
pT4 (*n* = 0)	0	0	
NA (*n* = 43)	38 (88.4%)	5 (11.6%)	
**Pathological N Stage (pN)**			
pNX (*n* = 11)	7 (63.6%)	4 (36.4%)	*0.043
pN0 (*n* = 40)	34 (85.0%)	6 (15.0%)	
pN1 (*n* = 21)	16 (76.2%)	5 (23.8%)	
pN2b (*n* = 12)	12 (100%)	0	
pN2c (*n* = 1)	0	1 (100%)	
NA (*n* = 43)	38 (88.4%)	5 (11.6%)	
**Pathological M Stage (pM)**			
pMX (*n* = 85)	69 (81.2%)	16 (18.8%)	0.299
NA (*n* = 43)	38 (88.4%)	5 (11.6%)	
**Stage (pTNM)**			
I (n = 30)	24 (80.0%)	6 (20.0%)	0.609
II (n = 17)	14 (82.4%)	3 (17.6%)	
III (*n* = 25)	19 (76.0%)	6 (24.0%)	
IV (*n* = 13)	12 (92.3%)	1 (7.7%)	
NA (*n* = 43)	38 (88.4%)	5 (11.6%)	
**Grade**			
G1 (*n* = 46)	40 (87.0%)	6 (13.0%)	0.524
G2 (*n* = 69)	56 (81.2%)	13 (18.8%)	
G3 (*n* = 9)	8 (88.9%)	1 (11.1%)	
G2-G3 (*n* = 2)	1 (50.0%)	1 (50.0%)	
NA (*n* = 2^¥^)	2 (100%)	0	
**Tumor Site**			
Tongue (*n* = 128)	107 (83.6%)	21 (16.4%)	—
**Tumor Greatest Dimension (cm)**			
mean ± SD	2.1 ± 1.1	2.3 ± 1.4	0.607
**Nodes Involved**			
mean ± SD	1.2 ± 2.0	0.8 ± 1.2	0.514

COL1A2, Collagen I alpha 2; CRNN, Cornulin; DCN, Decorin; TSCC, Tongue squamous cell carcinoma; SD, Standard deviation: NA, Not applicable because these are biopsy-only tongue tissues, for two patients grade not mentioned

a IRS ≤ +3

b IRS ≥ +4

*p*-value, Pearson’s chi-square test; pTNM and stage: As per AJCC guidelines; G1, Well differentiated TSCC; G2, Moderately differentiated TSCC; G3, Poorly differentiated TSCC

* Significant values (≤0.05)

**Supplementary Table S4.** Cardinal percentages of immunopositive epithelial cells IRS and keratinisation in TSCC whole slide images without categorising based on cutoff point. See https://figshare.com/s/35c73fc5606a17ae0c0c.

**Table 1.** Association between clinico-pathological features of TSCC and (A): COL1A2, (B): CRNN and (C): DCN Immunohistochemical staining.

A			
Pathologist 1	COL1A2 expression		*p*-value
	Low^a^	High^b^	
TSCC patients (*n* = 128)	99 (77.3%)	29 (22.7%)	—
**Age at diagnosis (years)**			
Mean ± SD	49.6 ± 13.1	48.8 ± 14.0	0.78
**Gender**			
Male (*n* = 85)	73 (85.9%)	12 (14.1%)	*0.001
Female (*n* = 43)	26 (60.5%)	17 (39.5%)	
**Pathological T stage (pT)**			
pT1 (*n* = 47)	37 (78.7%)	10 (21.3%)	0.589
pT2 (*n* = 30)	25 (83.3%)	5 (16.7%)	
pT3 (*n* = 8)	5 (62.5%)	3 (37.5%)	
pT4 (*n* = 0)	0	0	
NA (*n* = 43)	32 (74.4%)	11 (25.6%)	
**Pathological N stage (pN)**			
pNX (*n* = 11)	6 (54.5%)	5 (45.5%)	0.053
pN0 (*n* = 40)	34 (85.0%)	6 (15.0%)	
pN1 (*n* = 21)	14 (66.7%)	7 (33.3%)	
pN2b (*n* = 12)	12 (100%)	0	
pN2c (*n* = 1)	1 (100%)	0	
NA (*n* = 43)	32 (74.4%)	11 (25.6%)	
**Pathological M stage (pM)**			
pMX (*n* = 85)	67 (78.8%)	18 (21.2%)	0.574
NA (*n* = 43)	32 (74.4%)	11 (25.6%)	
**Stage (pTNM)**			
I (*n* = 30)	24 (80.0%)	6 (20.0%)	0.202
II (*n* = 17)	13 (76.5%)	4 (23.5%)	
III (*n* = 25)	17 (68.0%)	8 (32.0%)	
IV (*n* = 13)	13 (100%)	0	
NA (*n* = 43)	32 (74.4%)	11 (25.6%)	
**Grade**			
G1 (*n* = 46)	39 (84.8%)	7 (15.2%)	0.24
G2 (*n* = 69)	52 (75.4%)	17 (24.6%)	
G3 (*n* = 9)	6 (66.7%)	3 (33.3%)	
G2–G3 (*n* = 2)	1 (50.0%)	1 (50.0%)	
NA (*n* = 2^c^)	1 (50.0%)	1 (50.0%)	
**Tumour site**			
Tongue (*n* = 128)	99 (77.3%)	29 (22.7%)	^--^
**Tumour greatest dimension (cm)**			
Mean ± SD	2.2 ± 1.1	2.0 ± 1.4	0.465
**Nodes involved**			
Mean ± SD	1.3 ± 2.0	0.6 ± 0.8	0.197

B			
Pathologist 1	CRNN expression		*p*-value
	Low^a^	High^b^	
TSCC patients (*n* = 128)	67 (52.3%)	61 (47.7%)	^--^
**Age at diagnosis (years)**			
Mean ± SD	48.4 ± 13.0	50.5 ± 13.5	0.368
**Gender**			
Male (*n* = 85)	50 (58.8%)	35 (41.2%)	^*^0.039
Female (*n* = 43)	17 (39.5%)	26 (60.5%)	
**Pathological T stage (pT)**			
pT1 (*n* = 47)	22 (46.8%)	25 (53.2%)	0.061
pT2 (*n* = 30)	22 (73.3%)	8 (26.7%)	
pT3 (*n* = 8)	3 (37.5%)	5 (62.5%)	
pT4 (*n* = 0)	0	0	
NA (*n* = 43)	20 (46.5%)	23 (53.5%)	
**Pathological N stage (pN)**			
pNX (*n* = 11)	6 (54.5%)	5 (45.5%)	0.54
pN0 (*n* = 40)	21 (52.5%)	19 (47.5%)	
pN1 (*n* = 21)	10 (47.6%)	11 (52.4%)	
pN2b (*n* = 12)	9 (75.0%)	3 (25.0%)	
pN2c (*n* = 1)	1 (100%)	0	
NA (*n* = 43)	20 (46.5%)	23 (53.5%)	
**Pathological M stage (pM)**			
pMX (*n* = 85)	47 (55.3%)	38 (44.7%)	0.347
NA (*n* = 43)	20 (46.5%)	23 (53.5%)	
**Stage (pTNM)**			
I (*n* = 30)	14 (46.7%)	16 (53.3%)	0.253
II (*n* = 17)	11 (64.7%)	6 (35.3%)	
III (*n* = 25)	12 (48.0%)	13 (52.0%)	
IV (*n* = 13)	10 (76.9%)	3 (23.1%)	
NA (*n* = 43)	20 (46.5%)	23 (53.5%)	
**Grade**			
G1 (*n* = 46)	31 (67.4%)	15 (32.6%)	^*^0.02
G2 (*n* = 69)	29 (42.0%)	40 (58.0%)	
G3 (*n* = 9)	6 (66.7%)	3 (33.3%)	
G2–G3 (n = 2)	0	2 (100%)	
NA (n = 2^c^)	1 (50.0%)	1 (50.0%)	
**Tumour site**			
Tongue (*n* = 128)	67 (52.3%)	61 (47.7%)	^--^
**Tumour greatest dimension (cm)**			
Mean ± SD	2.4 ± 1.1	1.8 ± 1.2	^*^0.027
**Nodes involved**			
Mean ± SD	1.4 ± 2.1	0.9 ± 1.5	0.248

C			
Pathologist 1	DCN expression		*p*-value
	Low^a^	High^b^	
TSCC patients (*n* = 128)	112 (87.5%)	16 (12.5%)	^--^
**Age at diagnosis (years)**			
Mean ± SD	48.9 ± 13.0	52.4 ± 14.8	0.323
**Gender**			
Male (*n* = 85)	72 (84.7%)	13 (15.3%)	0.179
Female (*n* = 43)	40 (93.0%)	3 (7.0%)	
**Pathological T stage (pT)**			
pT1 (*n* = 47)	41 (87.2%)	6 (12.8%)	0.646
pT2 (*n* = 30)	27 (90.0%)	3 (10.0%)	
pT3 (*n* = 8)	6 (75.0%)	2 (25.0%)	
pT4 (*n* = 0)	0	0	
NA (*n* = 43)	38 (88.4%)	5 (11.6%)	
**Pathological N stage (pN)**			
pNX (*n* = 11)	8 (72.7%)	3 (27.3%)	0.127
pN0 (*n* = 40)	35 (87.5%)	5 (12.5%)	
pN1 (*n* = 21)	19 (90.5%)	2 (9.5%)	
pN2b (*n* = 12)	12 (100%)	0	
pN2c (*n* = 1)	0	1 (100%)	
NA (*n* = 43)	38 (88.4%)	5 (11.6%)	
**Pathological M stage (pM)**			
pMX (*n* = 85)	74 (87.1%)	11 (12.9%)	0.832
NA (*n* = 43)	38 (88.4%)	5 (11.6%)	
**Stage (pTNM)**			
I (*n* = 30)	25 (83.3%)	5 (16.7%)	0.973
II (*n* = 17)	15 (88.5%)	2 (11.8%)	
III (*n* = 25)	22 (88.0%)	3 (12.0%)	
IV (*n* = 13)	12 (92.3%)	1 (7.7%)	
NA (*n* = 43)	38 (88.4%)	5 (11.6%)	
**Grade**			
G1 (*n* = 46)	42 (91.3%)	4 (8.7%)	0.734
G2 (*n* = 69)	58 (84.1%)	11 (15.9%)	
G3 (*n* = 9)	8 (88.9%)	1 (11.1%)	
G2–G3 (*n* = 2)	2 (100%)	0	
NA (*n* = 2^c^)	2 (100%)	0	
**Tumour site**			
Tongue (*n* = 128)	112 (87.5%)	16 (12.5%)	^--^
**Tumour greatest dimension (cm)**			
Mean ± SD	2.1 ± 1.2	2.4 ± 1.2	0.375
**Nodes involved**			
Mean ± SD	1.2 ± 1.9	0.9 ± 1.5	0.653

COL1A2, Collagen I alpha 2; CRNN, Cornulin; DCN, Decorin; TSCC, Tongue squamous cell carcinoma; SD, Standard deviation; NA, Not applicable because these are biopsy-only tongue tissues; G1, Well differentiated TSCC; G2, Moderately differentiated TSCC; G3, Poorly differentiated TSCC

*p*-value, Pearson’s chi-square test

pTNM and stage: As per AJCC guidelines

a IRS ≤ +3

b IRS ≥ +4

c For two patients grade not mentioned

* significant values (≤ 0.05)

**Table 2.** Effect of addiction on TSCC. (A): COL1A2, (B): CRNN and (C): DCN (i) TSCC betel leaf addicts versus TSCC non-addiction, (ii) TSCC naswar addicts versus TSCC non-addiction, (iii) TSCC tobacco smoke addicts versus TSCC non-addiction.

A				
(i)				
**Pathologist 1**		**COL1A2 expression**		***p*-value**
		**Low^a^**	**High^b^**	
TSCC	Betel leaf addicts (*n* = 35)	26 (74.3%)	9 (25.7%)	0.418
	Non-addicts (*n* = 47)	31 (66.0%)	16 (34.0%)	
Total	82	57 (69.5%)	25 (30.5%)	
(ii)				
**Pathologist 1**		**COL1A2 expression**		***p*-value**
		**Low^a^**	**High^b^**	
TSCC	Naswar addicts (*n* = 13)	10 (76.9%)	3 (23.1%)	0.522
	Non-addicts (*n* = 47)	31 (66.0%)	16 (34.0%)	
Total	60	41 (68.3%)	19 (31.7%)	
(iii)				
**Pathologist 1**		**COL1A2 expression**		***p*-value**
		**Low^a^**	**High^b^**	
TSCC	Tobacco smoke addicts (*n* = 33)	32 (97.0%)	1 (3.0%)	*0.001
	Non-addicts (*n* = 47)	31 (66.0%)	16 (34.0%)	
Total	80	63 (78.8%)	17 (21.3%)	
**B**				
**(i)**				
**Pathologist 1**		**CRNN expression**		***p*-value**
		**Low^a^**	**High^b^**	
TSCC	Betel leaf addicts (*n* = 35)	20 (57.1%)	15 (42.9%)	0.091
	Non-addicts (*n* = 47)	18 (38.3%)	29 (61.7%)	
Total	82	38 (46.3%)	44 (53.7%)	
**(ii)**				
**Pathologist 1**		**CRNN expression**		***p*-value**
		**Low^a^**	**High^b^**	
TSCC	Naswar addicts (*n* = 13)	11 (84.6%)	2 (15.4%)	*0.003
	Non-addicts (*n* = 47)	18 (38.3%)	29 (61.7%)	
Total	60	29 (48.3%)	31 (51.7%)	
**(iii)**				
**Pathologist 1**		**CRNN expression**		***p*-value**
		**Low^a^**	**High^b^**	
TSCC	Tobacco smoke addicts (*n* = 33)	18 (54.5%)	15 (45.5%)	0.150
	Non-addicts (*n* = 47)	18 (38.3%)	29 (61.7%)	
Total	80	36 (45.0%)	44 (55.0%)	
**C**				
**(i)**				
**Pathologist 1**		**DCN expression**		***p*-value**
		**Low^a^**	**High^b^**	
TSCC	Betel leaf addicts (*n* = 35)	30 (85.7%)	5 (14.3%)	0.737
	Non-addicts (*n* = 47)	39 (83.0%)	8 (17.0%)	
Total	82	69 (84.1%)	13 (15.9%)	
**(ii)**				
**Pathologist 1**		**DCN expression**		***p*-value**
		**Low^a^**	**High^b^**	
TSCC	Naswar addicts (*n* = 13)	13 (100%)	0 (0.00%)	0.182
	Non-addicts (*n* = 47)	39 (83.0%)	8 (17.0%)	
Total	60	52 (86.7%)	8 (13.3%)	
(iii)				
**Pathologist 1**		**DCN expression**		***p*-value**
		**Low^a^**	**High^b^**	
TSCC	Tobacco smoke addicts (*n* = 33)	30 (90.9%)	3 (9.1%)	0.511
	Non-addicts (*n* = 47)	39 (83.0%)	8 (17.0%)	
Total	80	69 (86.3%)	11 (13.7%)	

COL1A2, Collagen I alpha 2; CRNN, Cornulin; DCN, Decorin; TSCC, Tongue squamous cell carcinoma*p*-value, Pearson’s chi-square test

a IRS ≤ +3

b IRS ≥ +4

* Significant values (≤ 0.05)

**Table 3.** Association between clinico-pathological features of non-cancer patients and (A): COL1A2, (B): CRNN and (C): DCN immunohistochemical staining.

A			
**Pathologist 1**	**COL1A2 expression**		***p*-value**
	**Low^a^**	**High^b^**	
Non-cancer patients (*n* = 48)	38 (79.2%)	10 (20.8%)	^--^
**Age at diagnosis (years)**			
Mean ± SD	43.0 ± 16.8	41.2 ± 14.5	0.765
**Gender**			
Male (*n* = 24)	18 (75.0%)	6 (25.0%)	0.477
Female (*n* = 24)	20 (83.3%)	4 (16.7%)	
**B**			
**Pathologist 1**	**CRNN expression**		***p*-value**
	**Low^a^**	**High^b^**	
Non-cancer patients (*n* = 48)	3 (6.2%)	45 (93.8%)	^--^
**Age at diagnosis (years)**			
Mean ± SD	43.0 ± 20.7	42.6 ± 16.2	0.964
**Gender**			
Male (*n* = 24)	2 (8.3%)	22 (91.7%)	1.000
Female (*n* = 24)	1 (4.2%)	23 (95.8%)	
**C**			
**Pathologist 1**	**DCN expression**		***p*-value**
	**Low^a^**	**High^b^**	
Non-cancer patients (*n* = 48)	44 (91.7%)	4 (8.3%)	^--^
**Age at diagnosis (years)**			
Mean ± SD	42.6 ± 16.5	42.8 ± 14.4	0.983
**Gender**			
Male (*n* = 24)	21 (87.5%)	3 (12.5%)	0.609
Female (*n* = 24)	23 (95.8%)	1 (4.2%)	

COL1A2, Collagen I alpha 2; CRNN, Cornulin; DCN, Decorin; SD, Standard deviation; *p*-value, Pearson’s chi-square test

a IRS ≤ +3

b IRS ≥ +4

significant values (≤0.05)

**Table 4.** Response for tested parameters in TSCC epithelial expression.

Tested parameters	COL1A2 *p*-value	CRNN *p*-value	DCN *p*-value
Pathological T stage (pT)	NS	NS	NS
Pathological N stage (pN)	NS	NS	NS
Pathological M stage (pM)	NS	NS	NS
Stage (pTNM)	NS	NS	NS
Grade	NS	S	NS
Tumour greatest dimension (cm)	NS	S	NS
Nodes involved	NS	NS	NS
Betel leaf addiction	NS	NS	NS
Naswar addiction	NS	S	NS
Tobacco smoke addiction	S	NS	NS

COL1A2, Collagen I alpha 2; CRNN, Cornulin; DCN, Decorin; TSCC, Tongue squamous cell carcinoma; S, significant *p*-values (≤0.05); NS, Non-significant *p*-values

Tables 1 and 2 *p*-values, Pearson’s chi-square test

pTNM and stage: As per AJCC guidelines
